# Effect of lipid metabolism disorder on liver function in patients with malignant tumors after chemotherapy: a case-control study

**DOI:** 10.1186/s12944-019-1063-y

**Published:** 2019-05-10

**Authors:** Yan Sun, Nie Zhang, Yun-Long Ding, Li-Jiang Yu, Jun Cai, De Ma, Wu Yang, Wang-Kun Lu, Jia-Li Niu

**Affiliations:** 1 Department of Oncology, JingJiang People’s Hospital, the Seventh Affiliated Hospital of Yangzhou University, Jiangsu, 214500 China; 2 Department of Endocrinology, JingJiang People’s Hospital, the Seventh Affiliated Hospital of Yangzhou University, Jiangsu, 214500 China; 3 Department of Neurology, JingJiang People’s Hospital, the Seventh Affiliated Hospital of Yangzhou University, Jiangsu, 214500 China; 4 Department of Clinical Pharmacy, JingJiang People’s Hospital, the Seventh Affiliated Hospital of Yangzhou University, No. 28, Zhongzhou Road, Jingjiang, Jiangsu, 214500 China

**Keywords:** Lipid metabolism, Tumor, Chemotherapy, Liver function

## Abstract

**Background:**

This study aims to investigate the effect of lipid metabolism disorder on liver function in patients with malignant tumors after chemotherapy.

**Method:**

A total of 428 patients with malignant tumors with normal liver function in our hospital between May 2013 to June 2018 were divided into an observation group (lipid metabolism disorder, *n* = 265) and control group (normal lipid metabolism, *n* = 163). The lipid metabolism levels and liver damage of the two groups were compared before and after chemotherapy.

**Results:**

No significant differences in age, gender, body mass index, tumor types, history of surgery, levels of alanine aminotransferase (ALT; an indicator of liver function), and chemotherapy regimen were observed between the two groups. However, the observation group showed increased levels of total cholesterol (*P* = 0.000), triglycerides (*P* = 0.000), and low-density lipoprotein (*P* = 0.01), as well as decreased levels of high-density lipoprotein (*P* = 0.000) before chemotherapy compared with the control group. Furthermore, patients with lipid metabolism disorders were more likely to develop abnormal liver function after chemotherapy. Moreover, mixed lipid metabolism disorder was more likely to cause severe liver damage after chemotherapy. Additionally, the number of patients with lipid metabolism disorders after chemotherapy (*n* = 367) was significantly increased compared with before chemotherapy (*n* = 265) (*P <* 0.01), indicating that chemotherapy might induce or aggravate an abnormal lipid metabolism.

**Conclusions:**

After receiving chemotherapy, patients with malignant tumors presenting lipid metabolism disorders are more prone to liver damage and lipid metabolism disorders than patients with a normal lipid metabolism.

## Background

Malignant tumors are a serious threat to human health, and chemotherapy remains one of the main treatments. However, chemotherapy has many toxic side effects [1]. For instance, as most chemotherapeutic drugs are metabolized by the liver, liver toxicity is one of the most common side effects [2]. Clinical manifestations of chemotherapy-induced liver damage are diverse, ranging from slight liver function abnormalities to severe cases of toxic hepatitis or fulminant hepatic failure. Hepatic toxicity is generally reversible [3], but may still cause fibrosis or cirrhosis if discontinued. In addition, liver damage caused by chemotherapeutic drugs may lead to lipid metabolism disorders and even hyperlipidemia [4, 5].

High-fat, high-energy diets have resulted in an increase in the incidence of lipid metabolism disorders [6]. Under this condition, reduced transport of fat causes lipids to accumulate in liver cells, resulting in a fatty liver [7]. Fat accumulation in the liver can affect the function of or destroy liver cells and cause connective tissue hyperplasia and cirrhosis, which affects drug metabolism [8]. Because chemotherapeutic drugs can cause liver damage and liver damage is associated with lipid metabolism disorders, we suspect that lipid metabolism disorders may be related to liver damage caused by chemotherapeutic drugs, but no research on this topic has been reported.

In the present study, we examined lipid metabolism in patients with malignant tumors who were treated at our hospital in recent years and assessed impairments in liver function after chemotherapy to investigate the effect of lipid metabolism disorders on liver function in patients undergoing chemotherapy.

## Materials and methods

### General information

This study included patients who were undergoing chemotherapy at our hospital from May 2013 to June 2018. Exclusion criteria were patients with diseases associated with liver damage, such as liver metastasis, viral hepatitis, drug-induced liver disease, total parenteral nutrition, and hepatolenticular degeneration. Inclusion criteria were patients diagnosed with malignant tumors via a pathological or cytological examination who required chemotherapy, had no chemotherapy contraindications, were aged≥18 years, had normal liver function (alanine aminotransferase (ALT) level ≤ 50 IU/L), and had a Kamofsky performance status ≥70 points. Before chemotherapy, tests of blood lipids and liver function were performed on all patients.

### Procedure

The blood lipid levels and liver function of each patient were assessed before chemotherapy. All indicators were in the normal range before chemotherapy. All patients underwent chemotherapy with a combination regimen according to the pathological type of malignancy. Common chemotherapy drugs included oxaliplatin, 5-fluorouracil, paclitaxel, cyclophosphamide, epirubicin, cisplatin, platinum, vinorelbine, gemcitabine, capecitabine, vincristine, and irinotecan, among others. Blood lipid levels and liver function were also measured approximately 1 week after chemotherapy was initiated. If abnormal liver function was observed during chemotherapy, chemotherapy was suspended and relevant drugs were administered as a protective treatment. Liver function was reassessed every 5–7 days until it returned to normal levels, after which chemotherapy was continued. After chemotherapy, liver function and blood lipid levels were analyzed again. In this study, liver function and blood lipid levels were all measured approximately 1 week after chemotherapy was initiated. Venous blood was collected in the morning from all patients who had fasted for more than 8 h, and total cholesterol (TC), triglyceride (TG), low-density lipoprotein cholesterol (LDL-C), and high-density lipoprotein cholesterol (HDL-C) levels were determined. Lipid indicators, liver function and ALT levels were assessed using enzymatic methods.

### Types of lipid metabolism disorders

Based on the recommendations for the prevention and treatment of dyslipidemia, lipid metabolism disorders are classified as follows [9]: 1. hypercholesterolemia (fasting serum TC level exceeding 5.72 mmol/L); 2. hypertriglyceridemia (fasting serum TG level exceeding 1.70 mmol/L); 3. mixed hyperlipidemia (increased fasting serum TC and TG levels); and 4. low high-density lipoproteinemia (fasting serum HDL level < 0.9 mmol/L).

### Criteria for evaluating liver function

According to the World Health Organization standards for the toxicity and side effects of anticancer drugs, the evaluation of liver function was defined using the following criteria: 1. normal liver function, serum ALT level ≤ 1.25 N (*N* = 40 IU/L); 2. liver function damage I level, 1.25 N < serum ALT level < 2.5 N; 3. liver function damage II level, 2.5 N < serum ALT level < 5 N; 4. liver function damage III level, 5 N < serum ALT level < 10 N; and 5. liver function damage IV level, serum ALT level > 10 N.

### Non-alcoholic fatty liver disease (NAFLD)

According to the guidelines for the diagnosis and treatment of NAFLD [10], the disease is divided into non-alcoholic simple fatty liver disease, non-alcoholic fatty liver hepatitis and non-alcoholic fatty liver cirrhosis.

### Statistical analysis

Statistical analyses were performed using SPSS 20.0 statistical analysis software. The count data are presented as means±standard deviations. T-tests were used for comparisons between groups. Dichotomous variables were analyzed using the χ^2^ test. If the number of observations was less than 5, Fisher’s exact probability test was used to calculate the *P* value. *P* < 0.05 was considered statistically significant.

## Results

### The observation group showed increased levels of TC, TG, and LDL and decreased levels of HDL before chemotherapy

A total of 428 patients with malignant tumors with normal liver function (199 males and 229 females, with an average age of 62.4 ± 9.2 years) were enrolled in our hospital from May 2013 to June 2018. These patients were divided into the lipid metabolism disorder group (observation group, *n* = 265) and normal lipid metabolism group (control group, *n* = 163) according to the lipid metabolism status before chemotherapy. In the observation group, 69 patients (26.0%) had high cholesterol levels, 98 patients (37.0%) had high TG levels, 61 patients (23.0%) had a mixed type, and 37 patients (14.0%) had a low HDL-C type. General characteristics of the two groups are shown in Table 1. No significant differences in age, gender, body mass index (BMI), tumor types, and history of surgery between the two groups.

**Table 1:** Comparison of the general conditions between the two groups $\left[\overline{x}\kern0.5em \pm \kern0.5em \mathrm{s}/\mathrm{case}\ \left(\%\right)\right]$

Item	Observation (*n* = 265)	Control (*n* = 163)	*P*
Age	62.0 ± 9.3	61.3 ± 9.0	0.247
Male	123 (46.4)	76 (46.6)	0.996
BMI	22.2 ± 2.0	21.8 ± 2.6	0.086
Tumor types			
Gastrointestinal cancer	105 (39.6)	61 (37.4)	0.650
Lung cancer	47 (17.7)	37 (22.7)	0.209
Breast cancer	56 (21.1)	32 (19.6)	0.709
Gynecologic cancer	25 (9.4)	16 (9.8)	0.896
Malignant lymphoma	10 (3.8)	6 (3.7)	0.961
Other tumors	22 (8.3)	11 (6.7)	0.559
Surgery	182 (68.7)	116 (71.2)	0.587
ALT before chemotherapy	26.2 ± 7.8	25.4 ± 9.4	0.361
TC before chemotherapy	5.5 ± 1.2	4.7 ± 0.7	0.000
TG before chemotherapy	1.8 ± 0.6	1.3 ± 0.2	0.000
HDL before chemotherapy	1.3 ± 0.5	1.4 ± 0.4	0.000
LDL before chemotherapy	3.2 ± 1.1	2.9 ± 0.8	0.010

*BMI* body mass index, *ALT* alanine aminotransferase, *TC* total cholesterol, *TG* triglycerides, *HDL* high-density lipid, *LDL* low-density lipoprotein; *P* < 0.05 was considered statistically significant

Furthermore, significant differences in ALT levels (a sensitive indicator of liver function) before chemotherapy were not observed between the observation (26.2 ± 7.8 IU/L) and control groups (25.4 ± 9.4 IU/L). However, the observation group showed increased levels of TC (*P* = 0.000), TG (*P* = 0.000), and LDL (*P* = 0.01) and decreased levels of HDL (*P* = 0.000) before chemotherapy compared with the control group. Moreover, significant differences in the chemotherapy regimen were not observed between the two groups (Table 2).

**Table 2:** Comparison of the chemotherapy regimens between the two groups [case(%)]

Item	Observation (*n* = 265)	Control (*n* = 163)	*P*
CHOP	10 (3.8)	6 (3.7)	0.961
CP	4 (1.5)	0(0)	0.302
DP	2 (0.8)	3 (1.8)	0.374
DVP	0 (0)	1 (0.6)	0.381
FOLFIRI	14 (5.3)	7 (4.3)	0.646
FOLFOX	76 (28.7)	39 (23.9)	0.281
GP	26 (9.8)	19 (11.7)	0.546
IP	2 (0.8)	0 (0)	0.527
NF	11 (4.2)	6 (3.7)	0.809
NP	39 (14.7)	30 (18.4)	0.314
PVB	0 (0)	2 (1.2)	0.144
PVD	2 (0.8)	2 (1.2)	0.637
TA	25 (9.4)	13 (8)	0.606
TF	6 (2.3)	9 (5.5)	0.075
TOX	2 (0.8)	0 (0)	0.527
TP	44 (16.6)	24 (14.7)	0.605
VA	0 (0)	2 (1.2)	0.144
VP	2 (0.8)	0 (0)	0.527

### The observation group showed a higher incidence of liver damage after chemotherapy

The levels of liver damage after chemotherapy in the two groups are shown in Table 3. The incidence of liver damage after chemotherapy was 47.2% in the observation group (125/ 265), with 111 patients presenting level I damage (41.9%), 8 patients presenting level II damage (3.0%), and 6 patients presenting level III damage (2.3%), whereas the incidence in the control group was 20.9% (34/163), with 29 presenting level I damage (17.8%), 3 patients presenting level II damage (1.8%), and 2 patients presenting level III damage (1.2%). The incidence of liver damage after chemotherapy in the observation group was significantly greater than in the control group (*P* < 0.01). Based on these results, patients with lipid metabolism disorder are more prone to liver damage after chemotherapy than patients with normal lipid metabolism.

**Table 3:** Comparison of liver function damage after chemotherapy between the two groups [case(%)]

Group	Level I	Level II	Level III	Level IV	Total
Observation (*n* = 265)	111 (41.9)	8 (3.0)	6 (2.3)	0 (0)	125 (47.2)^**^
Control (*n* = 163)	29 (17.8)	3 (1.8)	2 (1.2)	0 (0)	34 (20.9)

Compared with the control group,^*^P < 0.05, ^**^P < 0.01

### Types of lipid metabolism disorders before chemotherapy correlated with liver function after chemotherapy

The correlations between the types of lipid metabolism disorders before chemotherapy and liver function after chemotherapy are presented in Table 4. No significant differences in the percentages of patients with normal liver function and liver damage at levels I and level II were observed among the hypercholesterolemia group, hypertriglyceridemia group, the mixed hyperlipidemia group, and low-HDL group. However, liver function at damage level III after chemotherapy was observed in 6 patients in the mixed hyperlipidemia group, but was not observed in the other groups. Moreover, a significant difference in the percentage of patients presenting function damage level III after chemotherapy was observed among the four groups (*P* = 0.000). In addition, a statistically significant difference in the degree of liver damage after chemotherapy was observed among the four groups (*P* = 0.002). Thus, a significant correlation exists between the type of lipid metabolism disorder before chemotherapy and liver function after chemotherapy.

**Table 4:** Types of lipid metabolism disorders and liver function after chemotherapy [case (%)]

Type	Hypercholesterolemia	Hypertriglyceridemia	Mixed	Low-HDL	*P*
Normal	39 (56.5)	48 (49.0)	30 (49.2)	23 (62.2)	0.461
Level I	30 (43.5)	48 (49.0)	21 (34.4)	12 (32.4)	0.183
Level II	0 (0)	2 (2.0)	4 (6.6)	2 (5.4)	0.067
Level III	0 (0)	0 (0)	6 (9.8)	0 (0)	0.000
Total	69	98	61	37	0.002

### Changes in lipid metabolism before and after chemotherapy

In addition to investigating the effect of lipid metabolism disorders on damage to liver function in patients with cancer after chemotherapy, we also evaluated the effect of chemotherapy on lipid metabolism in patients. Two hundred sixty-five patients presented with dyslipidemia and 163 patients presented with normal lipid metabolism before chemotherapy. Interestingly, the number of patients with dyslipidemia (*n* = 367) after chemotherapy was significantly increased compared with before chemotherapy (*P <* 0.01) (Table 5). Based on these data, chemotherapy might induce or aggravate lipid metabolism disorders.

**Table 5:** Changes in lipid metabolism before and after chemotherapy

Lipid metabolism before chemotherapy	Lipid metabolism after chemotherapy		
	Disorder	Normal	Total
Disorders	239	26	265
Normal	128	35	163
Total	367	61	428

## Discussion

Clinically, many patients receiving chemotherapy develop lipid metabolism disorders. In addition, the administration of chemotherapy to patients with lipid metabolism disorders may increase the risk of liver damage. However, currently, evidence for the relationship between lipid metabolism disorders and liver damage after chemotherapy is limited. The results from the present study showed increased levels of TC, TG, and LDL and decreased levels of HDL in the patients with lipid metabolism disorder before chemotherapy compared with the patients with normal lipid metabolism (Table 1). Furthermore, patients with lipid metabolism disorders are more likely to develop abnormal liver function after chemotherapy (Table 3). Moreover, mixed lipid metabolism disorder is more likely to cause severe liver damage after chemotherapy (Table 4). In addition, the number of patients with lipid metabolism disorders after chemotherapy (*n* = 367) was significantly increased compared with before chemotherapy (*n* = 265) (Table 5), indicating that chemotherapy potentially induces or aggravates abnormal lipid metabolism.

According to the evidence, chemotherapy drugs can cause lipid metabolism disorders. Paclitaxel and platinum-based chemotherapy caused transient dyslipidemia in three patients with cancer [11]. The chemotherapy drug 5-fluorouracil induces steatosis by generating reactive oxygen species [12]. Lipid metabolism disorders may be induced by chemotherapy due to drug toxicity [13]. Consistent with this finding, chemotherapy induced or aggravated abnormal lipid metabolism in the present study, as indicated by the increased number of patients with lipid metabolism disorders after chemotherapy.

Chemotherapeutic drugs have been shown to induce liver damage [12, 14, 15]. Almost all chemotherapeutic drugs are metabolized by the liver, the metabolic center of the body, and thus easily lead to drug-induced liver damage. Chemotherapeutic drugs mainly cause liver damage mainly through the following three pathways: (1) drugs and their metabolites directly damage liver cells; (2) chemotherapy may aggravate liver damage in patients with pre-existing liver diseases; and (3) pre-existing liver diseases might reduce the activity of drug-metabolizing enzymes and prolong the duration of drug action, thereby increasing the toxicity of chemotherapeutic drugs.

As shown in recent studies, lipid metabolism is closely related to the occurrence and development of tumors. Patients with tumors tend to have lipid metabolism disorders. In the present study, compared with patients with normal lipid metabolism, patients with malignant tumors presenting dyslipidemia showed an increased incidence of hepatic impairment after chemotherapy. Thus, hepatotoxicity should be more carefully assessed when chemotherapeutic drugs are administered to patients with lipid metabolism disorders.

The mechanisms by which lipid metabolism disorders affect liver function after chemotherapy are not yet completely understood, but may be related to the factors described below. (1) Excess lipids directly exert adverse effects on liver function. When a lipid metabolism disorder occurs, the liver is unable to transport fat in a timely manner, thus causing fat to accumulate in liver cells and resulting in fatty liver. Fat accumulation in the liver affects liver function and destroys liver cells, subsequently reducing the liver compensatory capacity and causing connective tissue hyperplasia, cirrhosis, and liver damage [16]. (2) Pre-existing dyslipidemia indirectly contributes to liver damage after chemotherapy. Most chemotherapeutic drugs are cytotoxic and immunosuppressive agents, causing a certain degree of damage to the liver themselves [17]. Chronic liver damage is present in patients with lipid metabolism disorders, resulting in a decreased drug metabolism capacity and aggravation of liver damage. (3) Pro-inflammatory cytokines exacerbate liver damage. For example, tumor cell necrosis after chemotherapy causes the release of tumor necrosis factor, one of the main factors involved in liver cell damage and even hepatic necrosis [18]. (4) The oxidative stress response also contributes to liver damage. Excess intake of free fatty acids causes oxidative overload in the mitochondria of hepatocytes, producing more reactive oxygen species (ROS). The expression of uncoupling protein 2 is compensatorily increased to reduce ROS production, and a large amount of adenosine triphosphate is consumed, leading to the apoptosis or even necrosis of hepatocytes [19]. Hepatocyte necrosis produces a large amount of inflammatory factors, further increasing the production of free radicals.

Chemotherapy-induced liver damage in patients with cancer may occur during chemotherapy or after the total course. Although liver function impairment may be reversed in some patients after treatment, abnormal liver function may lead to an interruption of anti-tumor therapy and jeopardize the prognosis of patients [20]. The clinical consequences of liver damage after chemotherapy in patients with cancer vary. Mild cases may only produce an asymptomatic increase in ALT levels, spontaneous reversal may occur in some patients, and severe cases may involve jaundice, ascites, coagulation abnormalities, encephalopathy and other signs of liver failure, with a high mortality rate if not treated effectively [21]. The symptoms experienced by these patients are mild, with most having no symptoms or signs, and abnormalities are only observed when liver function is measured. For patients with symptoms and signs, except for jaundice, the main symptoms are fatigue, anorexia, bloating, abdominal pain, and fever. The clinical manifestations of patients are similar to the original disease; they are not specific and are difficult to identify. Thus, the clinical manifestations of liver damage after chemotherapy are subtle in some patients and may not be detected in a timely manner, with a risk of worsening adverse reactions during continued treatment. Therefore, transaminase levels should be regularly monitored in patients with cancer who are undergoing chemotherapy. Once abnormal liver function is detected, a hepatoprotective enzyme treatment should be administered in a timely manner to prevent the further aggravation of the disease and ensure the successful completion of chemotherapy.

Lipid metabolism disorders are major risk factors for cardiovascular and cerebrovascular diseases and exert important effects on the metastasis and prognosis of some tumors, particularly breast and gastrointestinal tumors. Therefore, blood lipid levels must be controlled in patients with tumors. Patients with cancer should pay special attention to their eating habits and avoid high-fat diets to prevent abnormal lipid metabolism. Routine screening of blood lipid and transaminase levels before chemotherapy is recommended for patients with cancer, as are preventive measures to regulate lipid metabolism and protect liver function to reduce the incidence of hepatic damage, liver failure and tumor-related mortality. Improvements in the quality of life of patients with cancer will have a positive impact on the prognosis.

## Conclusions

In conclusion, patients with malignant tumors presenting lipid metabolism disorders are more prone to liver damage after chemotherapy than patients with a normal lipid metabolism. Furthermore, mixed lipid metabolism disorders are more likely to cause severe liver damage.

## Abbreviations

ALT: Alanine aminotransferase
HDL-C: High-density lipid cholesterol
LDL-C: Low-density lipoprotein cholesterol
NAFLD: Non-alcoholic fatty liver disease
TC: Total cholesterol
TG: Triglycerides
